# Supplementary figures and images for: Exposure assessment of elemental carbon, polycyclic aromatic hydrocarbons and crystalline silica at the underground excavation sites for top-down construction buildings
Source: PLoS One. 2020 Sep 14;15(9):e0239010. doi: 10.1371/journal.pone.0239010 (PMC7489544; doi:10.1371/journal.pone.0239010)

**S1 Fig.** Target Monitoring Workplace (A~D)


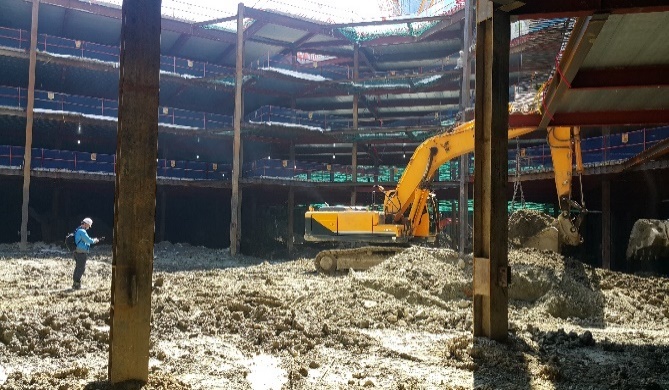

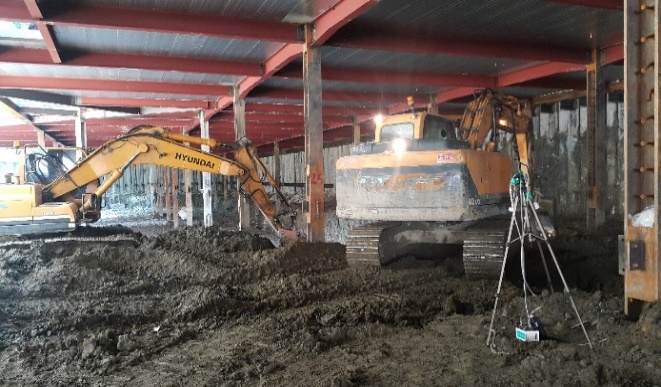


(Site A)


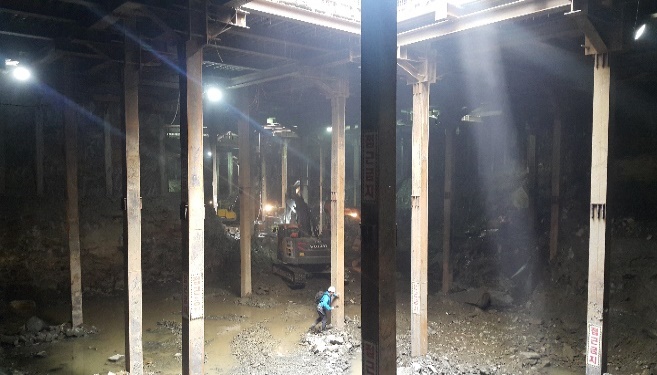

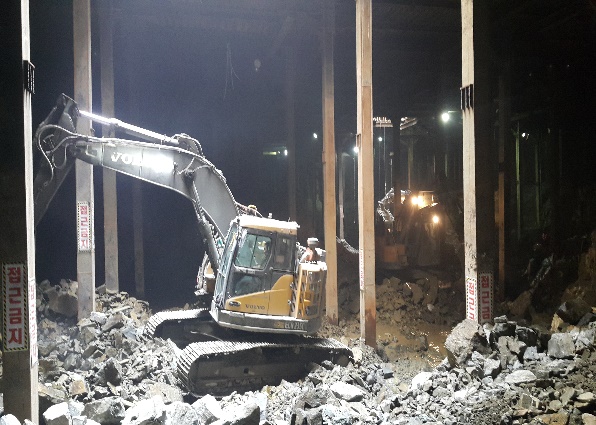


(Site B)


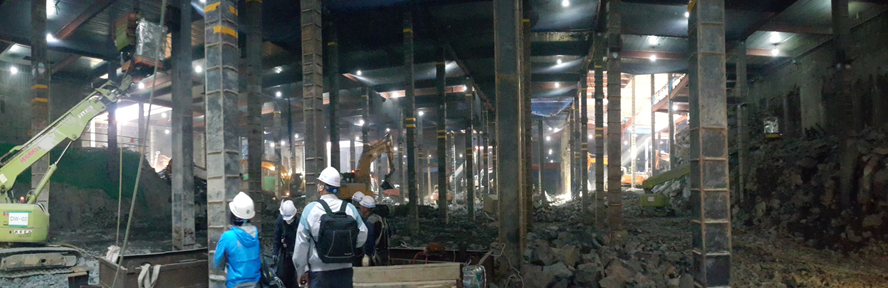


(Site C)


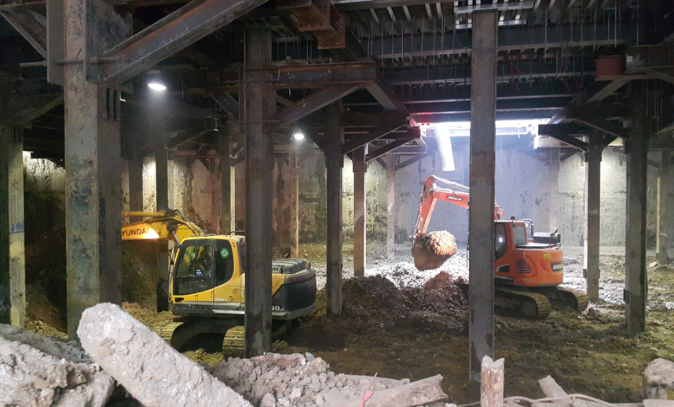

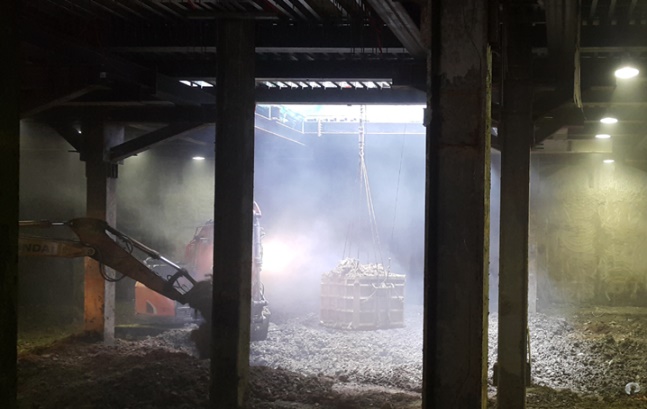


(Site D)

Supplement: S1 Fig — Target monitoring workplace (A~D). (DOCX) [file pone.0239010.s001.docx]
