# Supplementary material for: Exposure assessment of elemental carbon, polycyclic aromatic hydrocarbons and crystalline silica at the underground excavation sites for top-down construction buildings
Source: PLoS One. 2020 Sep 14;15(9):e0239010. doi: 10.1371/journal.pone.0239010 (PMC7489544; doi:10.1371/journal.pone.0239010)
Supplement: S1 Table — (DOCX) [file pone.0239010.s002.docx]

**S1 Table.** Analysis condition for OCEC Analyzer

| **Mode** | **Time (sec)** | **Temp. (℃)** |
| --- | --- | --- |
| Helium | 10 | - |
|  | 80 | 310 |
|  | 80 | 475 |
|  | 80 | 615 |
|  | 110 | 870 |
|  | 45 | 550 |
| Oxygen | 45 | 550 |
|  | 45 | 625 |
|  | 45 | 700 |
|  | 45 | 775 |
|  | 45 | 850 |
|  | 60 | 870 |
| Calibration | 110 | - |
