# Supplementary material for: Exposure assessment of elemental carbon, polycyclic aromatic hydrocarbons and crystalline silica at the underground excavation sites for top-down construction buildings
Source: PLoS One. 2020 Sep 14;15(9):e0239010. doi: 10.1371/journal.pone.0239010 (PMC7489544; doi:10.1371/journal.pone.0239010)
Supplement: S2 Table — (DOCX) [file pone.0239010.s003.docx]

**S2 Table.** Detection limits of PAHs species

| **PAHs species** | **Abbreviation** | **Chemical**  **Formula** | **MW, g/mol** | **Detection limits**  **(㎍/sample)** | **Toxic equivalent factors**  **(TEF)**  **[45]** | **Occupational exposure limits (OSHA)** |
| --- | --- | --- | --- | --- | --- | --- |
| Naphthalene | NAP | C_10_H_8_ | 128.17 | 0.409 | 0.001 | 10 ppm |
| Acenaphthylene | ACE | C_12_H_8_ | 152.2 | 0.291 | 0.001 | - |
| Acenaphthene | ACEN | C_12_H_10_ | 154.21 | 0.128 | 0.001 | - |
| Fluorene | FLUO | C_13_H_10_ | 166.22 | 0.112 | 0.001 | - |
| Phenanthrene | PHEN | C_14_H_10_ | 178.23 | 0.020 | 0.001 | 0.2 mg/㎥ |
| Anthracene | ANTH | C_14_H_10_ | 178.23 | 0.004 | 0.01 | 0.2 mg/㎥ |
| Fluoranthene | FLOUR | C_16_H_10_ | 202.26 | 0.026 | 0.001 | - |
| Pyrene | PYR | C_16_H_10_ | 202.26 | 0.063 | 0.001 | 0.2 mg/㎥ |
| Benz(a)anthracene | BAA | C_18_H_12_ | 228.29 | 0.006 | 0.1 | Suspected carcinogen |
| Chrysene | CHR | C_18_H_12_ | 228.29 | 0.048 | 0.01 | Animal Carcinogen |
| Benzo(b)fluoranthene | BBF | C_20_H_12_ | 252.32 | 0.016 | 0.1 | Suspected carcinogen |
| Benzo(k)fluoranthene | BKF | C_20_H_12_ | 252.32 | 0.040 | 0.1 | - |
| Benzo(a)pyrene | BAP | C_20_H_12_ | 252.32 | 0.015 | 1 | 0.2 mg/㎥,  Suspected carcinogen |
| Dibenz(a,h)anthracene | DIB | C_22_H_14_ | 278.35 | 0.423 | 0.1 | - |
| Benzo(ghi)perylene | GHI | C_22_H_12_ | 276.34 | 0.367 | 1 | - |
| Indeno(1,2,3-C,D)pyrene | IND | C_22_H_12_ | 276.34 | 0.052 | 0.01 | - |
