# Supplementary material for: Exposure assessment of elemental carbon, polycyclic aromatic hydrocarbons and crystalline silica at the underground excavation sites for top-down construction buildings
Source: PLoS One. 2020 Sep 14;15(9):e0239010. doi: 10.1371/journal.pone.0239010 (PMC7489544; doi:10.1371/journal.pone.0239010)
Supplement: S3 Table — (DOCX) [file pone.0239010.s004.docx]

**S3 Table.** Concentration of ***Total*** EC, OC, TC by construction site

(unit: ㎍/㎥)

| Type | Construction site | | EC | OC | TC | OC/EC (ratio) | |
| --- | --- | --- | --- | --- | --- | --- | --- |
| Inside  the vehicles | A | n | 5 | 5 | 5 | 5 | |
|  |  | AM±SD | 4.60±2.47 | 28.71±4.69 | 33.31±6.53 | 7.30±2.96 | |
|  |  | GM(GSD) | 4.17(1.618) | 28.41(1.174) | 32.83(1.207) | 6.82(1.516) | |
|  |  | Median | 3.83 | 26.61 | 30.90 | 6.95 | |
|  |  | Range | 2.69~8.70 | 23.78~34.93 | 26.47~43.64 | 4.01~11.46 | |
|  | B | n | 6 | 6 | 6 | 6 | |
|  |  | AM±SD | 9.83±2.53 | 61.71±47.13 | 71.54±46.56 | 6.74±5.38 | |
|  |  | GM(GSD) | 9.58(1.281) | 51.09(1.884) | 62.41(1.714) | 5.33(2.080) | |
|  |  | Median | 9.04 | 47.59 | 58.75 | 5.32 | |
|  |  | Range | 7.26~13.72 | 27.77~153.05 | 35.02~162.18 | 2.49~16.77 | |
|  | C | n | 6 | 6 | 6 | 6 | |
|  |  | AM±SD | 27.99±14.52 | 89.31±75.99 | 117.3±86.88 | 3.04±1.80 | |
|  |  | GM(GSD) | 25.07(1.674) | 66.98(2.234) | 94.19(2.032) | 2.67(1.714) | |
|  |  | Median | 25.15 | 44.91 | 72.62 | 2.52 | |
|  |  | Range | 12.89~52.22 | 33.89~192.81 | 50.77~233.66 | 1.35~16.77 | |
|  | D | n | 6 | 6 | 6 | 6 | |
|  |  | AM±SD | 9.55±6.94 | 40.66±14.28 | 50.2±20.8 | 5.90±3.21 | |
|  |  | GM(GSD) | 7.30(2.346) | 38.48(1.447) | 46.44(1.555) | 5.27(1.671) | |
|  |  | Median | 7.82 | 41.41 | 50.05 | 5.49 | |
|  |  | Range | 2.09~19.59 | 24.57~54.62 | 26.66~74.21 | 2.79~11.76 | |
| *ANOVA test* | | | *p<0.001* | *p=0.103* | *p<0.05* | *p=0.064* | |
| Outside  the vehicles | A | n | 5 | 5 | 5 | | 5 |
|  |  | AM±SD | 4.84±1.63 | 16.44±4.05 | 21.29±4.40 | 3.69±1.59 | |
|  |  | GM(GSD) | 4.65(1.363) | 15.95(1.343) | 20.85(1.268) | 3.43(1.529) | |
|  |  | Median | 4.30 | 17.27 | 23.09 | 3.84 | |
|  |  | Range | 3.24~7.55 | 9.55~19.85 | 13.85~24.81 | 2.22~6.13 | |
|  | B | n | 8 | 8 | 8 | 8 | |
|  |  | AM±SD | 61.98±7.98 | 116.98±12.73 | 178.97±17.73 | 1.90±0.24 | |
|  |  | GM(GSD) | 61.53(1.138) | 116.38(1.114) | 178.21(1.103) | 1.89(1.137) | |
|  |  | Median | 63.37 | 114.88 | 173.48 | 1.84 | |
|  |  | Range | 52.53~74.32 | 101.04~137.85 | 156.03~204.07 | 1.53~2.20 | |
|  | C | n | 9 | 9 | 9 | 9 | |
|  |  | AM±SD | 89.30±33.51 | 93.84±41.0 | 183.13±73.04 | 1.10±0.25 | |
|  |  | GM(GSD) | 78.18(1.968) | 83.88(1.751) | 162.87(1.829) | 1.07(1.236) | |
|  |  | Median | 91.3 | 84.11 | 176.38 | 1.05 | |
|  |  | Range | 13.65~132.48 | 22.55~171.48 | 36.2~303.97 | 0.82~1.65 | |
|  | D | n | 7 | 7 | 7 | 7 | |
|  |  | AM±SD | 21.95±8.80 | 63.63±41.73 | 85.58±48.51 | 2.94±1.19 | |
|  |  | GM(GSD) | 19.11(1.985) | 51.49(2.078) | 72.02(1.983) | 2.69(1.613) | |
|  |  | Median | 24.91 | 42.40 | 62.69 | 3.67 | |
|  |  | Range | 4.22~31.45 | 16.71~127.19 | 20.93~158.63 | 1.25~4.04 | |
| *ANOVA test* | | | *p<0.001* | *p<0.001* | *p<0.001* | *p<0.001* | |
