# Supplementary material for: Exposure assessment of elemental carbon, polycyclic aromatic hydrocarbons and crystalline silica at the underground excavation sites for top-down construction buildings
Source: PLoS One. 2020 Sep 14;15(9):e0239010. doi: 10.1371/journal.pone.0239010 (PMC7489544; doi:10.1371/journal.pone.0239010)
Supplement: S4 Table — (DOCX) [file pone.0239010.s005.docx]

**S4 Table.** Concentration of ***respirable*** EC, OC, TC by construction site

(unit: ㎍/㎥)

| Type | Construction site | | EC | OC | | TC | OC/EC (ratio) |
| --- | --- | --- | --- | --- | --- | --- | --- |
| Inside  the vehicles | A | n | 6 | 6 | 6 | | 6 |
|  |  | AM±SD | 4.68±2.47 | 25.06±4.16 | 29.73±4.01 | | 6.74±4.00 |
|  |  | GM(GSD) | 4.23(1.616) | 24.77(1.182) | 29.5(1.15) | | 5.86(1.784) |
|  |  | Median | 4.25 | 24.98 | 29.50 | | 5.26 |
|  |  | Range | 2.34~9.30 | 19.11~31.62 | 23.42~34.12 | | 2.67~13.51 |
|  | B | n | 6 | 6 | 6 | | 6 |
|  |  | AM±SD | 10.29±2.82 | 50.13±36.99 | 60.42±35.84 | | 5.49±4.87 |
|  |  | GM(GSD) | 9.99(1.293) | 42.39(1.802) | 54.11(1.617) | | 4.24(2.102) |
|  |  | Median | 8.70 | 37.55 | 48.38 | | 3.98 |
|  |  | Range | 8.23~14.43 | 23.4~122.99 | 31.84~131.21 | | 2.03~14.95 |
|  | C | n | 5 | 5 | 5 | | 5 |
|  |  | AM±SD | 31.28±17.83 | 82.5±76.81 | 113.78±89.64 | | 2.50±1.93 |
|  |  | GM(GSD) | 27.41(1.784) | 54.94(2.769) | 86.24(2.324) | | 2.00(2.086) |
|  |  | Median | 29.3 | 31.10 | 68.45 | | 1.81 |
|  |  | Range | 13.87~58.43 | 23.07~166.90 | 39.01~225.33 | | 0.83~5.67 |
|  | D | n | 6 | 6 | 6 | | 6 |
|  |  | AM±SD | 7.83±5.64 | 26.28±4.03 | 34.11±9.08 | | 5.51±4.35 |
|  |  | GM(GSD) | 5.97(2.381) | 26.02(1.166) | 33.08(1.315) | | 4.36(2.096) |
|  |  | Median | 6.69 | 25.68 | 34.31 | | 4.27 |
|  |  | Range | 1.57~16.4 | 21.33~31.89 | 22.90~45.83 | | 1.80~13.56 |
| *ANOVA test* | | | *p<0.001* | *p=0.086* | *p<0.01* | | *p=0.115* |
| Outside  the vehicles | A | n | 6 | 6 | | 6 | 6 |
|  |  | AM±SD | 4.82±1.99 | 13.92±3.56 | | 18.74±4.04 | 3.23±1.41 |
|  |  | GM(GSD) | 4.54(1.437) | 13.51(1.321) | | 18.34(1.266) | 2.97(1.571) |
|  |  | Median | 4.08 | 13.64 | | 19.39 | 2.99 |
|  |  | Range | 3.15~8.53 | 8.33~18.68 | | 12.21~22.96 | 1.62~5.28 |
|  | B | n | 9 | 9 | | 9 | 9 |
|  |  | AM±SD | 60.35±11.66 | 61.27±13.14 | | 121.61±22.0 | 1.03±0.20 |
|  |  | GM(GSD) | 59.34(1.217) | 59.95(1.251) | | 119.73(1.21) | 1.01(1.201) |
|  |  | Median | 58.88 | 58.73 | | 125.95 | 0.99 |
|  |  | Range | 43.15~79.01 | 40.27~77.22 | | 83.42~156.23 | 0.77~1.45 |
|  | C | n | 9 | 9 | | 9 | 9 |
|  |  | AM±SD | 119.7±35.13 | 82.08±25.05 | | 201.78±59.64 | 0.68±0.06 |
|  |  | GM(GSD) | 115.72(1.307) | 78.83(1.35) | | 194.70(1.32) | 0.68(1.090) |
|  |  | Median | 103.94 | 82.27 | | 186.21 | 0.65 |
|  |  | Range | 83.66~191.42 | 52.26~123.54 | | 135.92~314.96 | 0.62~0.79 |
|  | D | n | 6 | 6 | | 6 | 6 |
|  |  | AM±SD | 17.77±7.31 | 29.73±11.05 | | 47.5±16.27 | 2.03±1.22 |
|  |  | GM(GSD) | 15.62(1.919) | 28.16(1.425) | | 44.84(1.476) | 1.80(1.669) |
|  |  | Median | 19.56 | 24.87 | | 46.10 | 1.68 |
|  |  | Range | 4.28~25.36 | 18.68~46.12 | | 22.96~67.17 | 1.02~4.36 |
| *ANOVA test* | | | *p<0.001* | *p<0.001* | | *p<0.001* | *p<0.001* |
