# Supplementary material for: Exposure assessment of elemental carbon, polycyclic aromatic hydrocarbons and crystalline silica at the underground excavation sites for top-down construction buildings
Source: PLoS One. 2020 Sep 14;15(9):e0239010. doi: 10.1371/journal.pone.0239010 (PMC7489544; doi:10.1371/journal.pone.0239010)
Supplement: Attachment — Submitted filename: Response to Reviews_PONE-D-20-13027.doc [file pone.0239010.s006.doc]

**Response to Reviewers**

**▶ *Manuscript ID*: PONE-D-20-13027**

**▶ *Title*: Exposure Assessment of Elemental Carbon, Polycyclic Aromatic Hydrocarbons and Crystalline Silica at the Underground Excavation Sites for Top-Down Construction Buildings**

▶ *Authors*: Hyunhee Park, Eunsong Hwang, Miyeon Jang, Chungsik Yoon

**Reviewer 1.**

**1.1. General Comments**

The paper deals with a subject that falls within the scope of the journal, but the manuscript needs big improvements concerning the overall presentation. Overall, the article should be written more clearly..

Thank you very much for your comments and constructive suggestions. I answered your specific questions and comments point-by-point below. Most of your suggestions are reflected in the revised manuscript, and are detailed in the following section.

**1.2. Specific Question**

**(1)**

***Comment*:** There is no abstract in the work. This is unacceptable.

***Section***: Abstract

***Response*:** When we first submitted the manuscript, we also submitted the abstract, but I wonder why there wasn’t. Anyway, the first abstract had divided into four subheadings before, i.e. objective, method, result and conclusions. It is revised to combine all four sections under one heading “abstract” without any subheading. Please see below

***Original manuscript:***

Abstract

Objective

Enclosed underground excavation worksite has an environment with poor ventilation and exposure to hazardous substances from diesel engine exhaust and construction materials. The objective of this study was to evaluate the exposure level of elemental carbon (EC), organic carbon (OC), total carbon (TC), polycyclic aromatic hydrocarbons (PAHs), dust and crystalline silica (CS) during underground excavation work for top down construction buildings.

Method

Active local air sampling for EC, OC, and TC (n=105), PAHs (n=50) and CS (n=34) was conducted from inside and outside the excavator at underground excavation workshop in four different construction sites. EC, OC, TC and CS were sampled with each respirable and total particulates. EC, OC, and TC were collected on quartz-filter and analyzed using the thermal optical transmittance method. PAHs was collected on polytetrafluorethylene filter with XAD-2 and analyzed using liquid chromatography with fluorescence detector. CS and particulates were collected on poly vinyl chloride filter and analyzed using fourier-transform infrared spectroscopy.

Result

The geometric mean of respirable EC, total PAHs, and respirable CS were 8.69 ㎍/㎥, 6.818 ㎍/㎥ and 0.017 mg/㎥ from inside the excavator and 33.20 ㎍/㎥, 3.934 ㎍/㎥ and 0.079 mg/㎥ from outside the excavator (underground excavation workshop), respectively. The EC and RCS concentration from outside the excavator is significantly higher than that of inside the excavator (p<0.01). The worksite with rock ground, higher vehicle density, blasting and enclosed environments had higher exposure to EC than other sites (p<0.05). There was no significant difference of EC concentration between total and respirable particulates. In top down construction sites, EC concentrations during underground excavation work exceeded recommended exposure limits as 20 ㎍/㎥, accounted for about 50% of the total sample, and the level of concentration of RCS exceeded the 1.5 times of occupational exposure limit, 0.05 mg/㎥.

Conclusion

We assessed underground excavation worker’s exposure to EC, OC, TC, PAHs and CS. Efforts are needed to minimize exposure to diesel engine exhaust and silica in underground excavation sites. Management of diesel engine vehicle, supply of fresh air and ventilation and introducing water facilities to create wet environment in underground worksites are strongly suggested.

***Revised manuscript:***

Abstract

Enclosed underground excavation worksite has an environment with poor ventilation and exposure to hazardous substances from diesel engine exhaust and construction materials . The objective of this study was to evaluate the exposure level of elemental carbon (EC), organic carbon (OC), total carbon (TC), polycyclic aromatic hydrocarbons (PAHs), dust and crystalline silica (CS) during underground excavation work for top down construction buildings. Active local air sampling for EC, OC, and TC (n=105), PAHs (n=50) dust (n=34) and CS (n=34) was conducted from inside and outside the excavator at underground excavation workshop in four different construction sites. EC, OC, TC and CS were sampled with each respirable and total particulates. EC, OC, and TC were collected on quartz-filter and analyzed using the thermal optical transmittance method. PAHs was collected on polytetrafluorethylene filter with XAD-2 and analyzed using liquid chromatography with fluorescence detector. CS and particulates were collected on poly vinyl chloride filter and analyzed using fourier-transform infrared spectroscopy. The geometric mean of respirable EC, OC, TC, total PAHs, respirable dust and respirable CS were 8.69 ㎍/㎥, 34.32 ㎍/㎥, 44.96 ㎍/㎥, 6.818 ㎍/㎥ 0.13 mg/㎥ and 0.02 mg/㎥ from inside the excavator and 33.20 ㎍/㎥, 46.53 ㎍/㎥, 78.21 ㎍/㎥, 3.934 ㎍/㎥, 0.9 mg/㎥ and 0.08 mg/㎥ from outside the excavator (underground excavation workshop), respectively.. The EC and RCS concentration from outside the excavator is significantly higher than that of inside the excavator (p<0.01). The worksite with rock ground, higher vehicle density, blasting and enclosed environments had higher exposure to EC than other sites (p<0.05). There was no significant difference of EC concentration between total and respirable particulates. In top down construction sites, EC concentrations during underground excavation work exceeded recommended exposure limits as 20 ㎍/㎥, accounted for about 50% of the total sample, and the level of concentration of RCS exceeded the 1.5 times of occupational exposure limit, 0.05 mg/㎥. We assessed underground excavation worker’s exposure to EC, OC, TC, PAHs and CS. Efforts are needed to minimize exposure to diesel engine exhaust and silica in underground excavation sites. Management of diesel engine vehicle, supply of fresh air and ventilation and introducing water facilities to create wet environment in underground worksites are strongly suggested.

(***Manuscript Page 2~3, Line 20-52)***

**(2)**

***Comment*:** The introduction section is poorly organized, it is good to express the need for the study with the backlog of literature exists in the framework. There is no background in this introduction stating the urge and novelty of the study in which innovative ideas must be flown through the background along with the useful insights The introduction must be improved. In introduction, please review the previous studies. What is the novelty for this article compared with existing studies?

***Section***: Introduction

***Response*:** In the introduction section, some previous similar literature were described and the novelty of our work was highlighted in relation to the previous studies.

***Original manuscript:***

Newly added

***Revised manuscript:***

However, the concentration levels and the workers’ exposure to RCS and DEE in construction sites that employ top-down excavation methods has yet to be reported. Previous studies have reported the concentration of RCS in construction sites based on typical occupations of tunnel construction worker [4-6], cement mason and concrete finisher [5-8], and building demolition [5, 9-11]. The concentration of DEE in construction sites were reported for tunnel [4, 12-13] and highway [14]. (***Manuscript Page 4, Line 69-74)***

**(3)**

***Comment*:** The problem of dusting occurs in many industries. You also have to write about it, e.g. <https://doi.org/10.3390/ijerph15091846>

***Section***: Discussion

***Response*:** The results of dust exposure in this study were compared with the results of dust exposure in other studies by various types of construction sites.

***Revised manuscript:***

Newly added

***Revised manuscript:***

The concentration of TD and RD from outside the excavators was 10 times higher than that of inside the excavators. Brodny and Tutak [54] reported the level of harmful dusts on fully powered longwall coal mines and the research results showed that type of activities and working location had a significant effect on the level of dust exposed. The concentration of RD from outside the excavators was similar with laborer(2.46 mg/㎥) and bricklayer (2.13 mg/㎥) [9] and much lower than that of painter blaster(13.50 mg/㎥) [9], tuck pointer(15.40 mg/㎥) [7], recess miller(5.08 mg/㎥) and demolition workers(23.67 mg/㎥) [8]. Dust showed a strong correlation with the crystalline silica concentration. ***(Manuscript Page 34, Line 432~439)***

**(4)**

***Comment*:** The structure of the paper should include the following sections: introduction, theoretical framework, materials method, results, discussion and conclusions.

***Section***: Introduction

***Response*:**

The theoretical framework was not an essential component in the manuscript organization of PLOS ONE, so the contents were included in the introduction.

***Revised manuscript:***

Newly added

***Revised manuscript:***

However, the problem is that there are no reported data on how high concentrations of crystalline silica, diesel engine exhaust and dust may be exposed to workers working in underground excavation workshops due to insufficient ventilation .

Thus the research questions of present study intends to evaluate the concentration of EC and PAHs (as representative indicators of DEE), RCS, and other respirable particulates to which workers are exposed in underground excavation sites employing top-down construction methods, and also evaluate the correlations between these hazardous substances

***(Manuscript Page 6, Line 98-105)***

**(5)**

***Comment*:** Need strong comment on scientific outcomes

***Section***: Discussion and Conclusions

***Response*:** The significance of our outcomes highlighted in conclusion parts..

***Revised manuscript:***

The concentrations of EC, OC, and TC were evaluated to determine the exposure level of DEE in the underground excavation workshop employing top-down construction methods

***Revised manuscript:***

The purpose of the study, the evaluation of DEE, RCS and respiratory dust in underground excavation sites, was well accomplished by appropriate evaluation methods ***(Manuscript Page 28, Line 326-328)***

Workers working in underground excavation workshops were exposed to high concentrations of crystalline silica, diesel engine exhaust that could have significant adverse health effect. Therefore, engineering and managerial improvement necessary to improve the working environment in underground excavation workshop.. ***(Manuscript Page 36, Line 452-455)***

Through this study, to improve the working environment and prevent occupational diseases in underground excavation workers, the results of the evaluation of the hazards were derived, and this data might be used for preparing engineering and managerial improvement measures.

***(Manuscript Page 36, Line 460-463)***

**Reviewer 2.**

**2.1. General Comments**

I think the title accurately reflects the purpose of the paper.

The abstract is concise and informative in concerning to matter under investigation, research method adopted and main results and conclusions.

The paper achieves its declared purpose and was well structured and thought out logically.

The English and syntax are correct.

In general, it is possible to state that the manuscript is technically sound, and do the data support the conclusions, and also authors made all data underlying the findings in their manuscript fully available.

The methodology and main research options are summarily referred in abstract and little explored in introduction section. A greater emphasis must be given this matter.

Thank you very much for your comments and constructive suggestions. I answered your questions and comments point-by-point below. Most of your suggestions are reflected in the revised manuscript, and are detailed in the following section.

**2.2 Specific Comments**

**(1)**

***Comment*:** The general research method seems to have been rigorous, clear and properly accounted for. However, some options adopted in concerning to research method and resources used are not adequately explained and defended. For example, options related to statistical analysis should be discussed; and Sampling is not sufficiently clear and defended.

***Section***: Materials and methods

***Response*:** A description of the number of samples collected and the statistical analysis method was added.

***Original manuscript:***

The EC and PAHs were assessed for 2 to 3 days at each of the four sites, and total dust, respirable dust and RCS were assessed for 1 day at three sites. For each site, measurements were carried out in three areas inside the excavator cabs and three areas of outside the excavator in underground workshops

***Revised manuscript:***

At each of four construction sites, EC and PAHs were assessed for two days at three locations inside and outside the excavator. In addition, at the construction sites B and C, EC, PAHs and total dust, respirable dust and RCS were assessed outside the excavator and at construction site D, total dust, respirable dust and RCS were assessed both inside and outside the excavator for two days. Therefore, 24 EC(each total and respirable), 24 PAHs, and 6 RCS were collected inside the excavator, and 30 EC(each total and respirable), 30 PAHs, and 11 RCS were collected from the outside of the excavator ***(Manuscript page 7~8, Line 124-130)***

Evaluation groups did not follow the normal or log-normal distribution. However, the form of the data is close to the lognormal distribution ***(Manuscript page 13, Line 196-197)***

**(2)**

***Comment*:** The criteria considered for the selection of the projects (construction sites) are not explained neither discussed. The author(s) should improve and clarify significantly this point.

***Section*:** Materials and methods

***Response*:**

The background of the selection of the research site was described.

***Original manuscript:***

Newly added

***Revised manuscript:***

The evaluation target groups were sites that were constructed by applying the top-down construction method, and voluntarily agreed to the evaluation. The sites was selected so that sites with diverse types of ground, including soft rock, hard rock, and soil. ***(Manuscript page 7), Line 113-115)***

**(3)**

***Comment*:** The references are up to date and represent adequately the previous work and the paper addresses the key issues encompassed by the topic.

Tables and figures are elucidative and help to understand. However all figures should include the axes titles as well as the units in axes. .

***Section***: Results

***Response*:** We agree with the reviwer’s opinion

***Original manuscript:***

-

***Revised manuscript:***

**Figure 1.** Mean concentration of EC, OC and TC, along with standard deviation (error bar), for each total and respirable particulates from inside and outside the excavator in underground excavation site

***(Manuscript page 17, Line 239)***

**(4)**

***Comment*:** Don’t use the first verbal person. Please remove "We", or "Our" throughout the ALL text

***Section***: Abstract

***Response*:** ‘We’ was removed

***Original manuscript:***

We assessed underground excavation worker’s exposure to EC, OC, TC, PAHs and CS.

***Revised manuscript:***

The concentration of EC, OC, TC, PAHs, dust and CS was assessed for underground excavation worker. ***(Manuscript page 3, Line 48-49)***

**(5)**

***Comment*:** In conclusions or discussion sections, I think it would be better to include a small discussion / analysis on whether the results and findings of this paper corresponded to the objectives of the study and will contribute to the increase in knowledge in the studied domain and to the better performance of industry. What were the main contributions from this study? And what is the use of the findings for future studies?

***Section***: Conclusions

***Response*:** The sentence which described the main contributions from this study and further studies is added

***Original manuscript:***

Newly added

***Revised manuscript:***

Workers working in underground excavation workshops were exposed to high concentrations of crystalline silica, diesel engine exhaust that could have significant adverse health effect. Therefore, engineering and managerial improvement necessary to improve the working environment in underground excavation workshop***. (Manuscript Page 36, Line 452-455)***

Through this study, we intend to use it to improve the working environment and prevent occupational diseases in underground excavation workers. Research data could be used for further studies to prepare engineering and managerial improvement measures.

***(Manuscript page 36~37, Line 460-463)***
