# Supplementary material for: Exposure assessment of elemental carbon, polycyclic aromatic hydrocarbons and crystalline silica at the underground excavation sites for top-down construction buildings
Source: PLoS One. 2020 Sep 14;15(9):e0239010. doi: 10.1371/journal.pone.0239010 (PMC7489544; doi:10.1371/journal.pone.0239010)
Supplement: Attachment — Submitted filename: Response to Reviews_PONE-D-20-13027_20200831.doc [file pone.0239010.s007.doc]

**Response to Reviewers(2^nd^ revision)**

**▶ *Manuscript ID*: PONE-D-20-13027**

**▶ *Title*: Exposure Assessment of Elemental Carbon, Polycyclic Aromatic Hydrocarbons and Crystalline Silica at the Underground Excavation Sites for Top-Down Construction Buildings**

▶ *Authors*: Hyunhee Park, Eunsong Hwang, Miyeon Jang, Chungsik Yoon

**# linguistic issues.**

Thank you very much for your comments and constructive suggestions. I answered your specific questions and comments point-by-point below. Most of your suggestions are reflected in the revised manuscript, and are detailed in the following section.

**(1)**

***Comment*:** Remove “the” before “1.5 times” at line 45 and remove the sentence “The concentration of EC, OC, TC, PAHs , dust and CS was assessed for underground excavation worker” (under sub-headings, this sentence works but not in a combined abstract) at lines 48-49. This has already been mentioned.

Introduction.

***Section***: Abstract

***Response*:** “the” before “1.5 times” and the sentence “The concentration of EC, OC, TC, PAHs , dust and CS was assessed for underground excavation worker” was removed

***Original manuscript:***

In top down construction sites, EC concentrations during underground excavation work exceeded recommended exposure limits as 20 ㎍/㎥, accounted for about 50% of the total sample, and the level of concentration of RCS exceeded the 1.5 times of occupational exposure limit, 0.05 mg/㎥. The concentration of EC, OC, TC, PAHs, dust and CS was assessed for underground excavation worker. Efforts are needed to minimize exposure to diesel engine exhaust and silica in underground excavation sites.

***Revised manuscript:***

In top down construction sites, EC concentrations during underground excavation work exceeded recommended exposure limits as 20 ㎍/㎥, accounted for about 50% of the total sample, and the level of concentration of RCS exceeded 1.5 times of occupational exposure limit, 0.05 mg/㎥. Efforts are needed to minimize exposure to diesel engine exhaust and silica in underground excavation sites. (***Manuscript Page 3, Line 42-43)***

**(2)**

***Comment*:** You have now mentioned the novelty of the work, but the novelty as expressed at line 69 needs to be expressed in a better linguistic manner, like, “Although, the concentration levels and workers’ exposure to contaminants/pollutants in tunnel and highway construction have been reported by many researchers [Ref….], its evaluation for excavation works in top-down constructions is still missing”.

***Section***: Introduction

***Response*:** The sentence was expressed in a better linguistic manner as recommended.

***Original manuscript:***

However, the concentration levels and the workers’ exposure to RCS and DEE in construction sites that employ top-down excavation methods has yet to be reported. Previous studies have reported the concentration of RCS in construction sites based on typical occupations of tunnel construction worker [4-6], cement mason and concrete finisher [5-8], and building demolition [5, 9-11]. The concentration of DEE in construction sites were reported for tunnel [4, 12-13] and highway [14].

***Revised manuscript:***

Previous studies have reported the concentration of RCS in construction sites based on typical occupations of tunnel construction worker [4-6], cement mason and concrete finisher [5-8], and building demolition [5, 9-11]. The concentration of DEE in construction sites were reported for tunnel [4, 12-13] and highway [14]. Although, the concentration levels and workers’ exposure to contaminants in tunnel and highway construction have been reported by many researchers, its evaluation for excavation works in top-down constructions is still missing (***Manuscript Page 4, Line 69-71)***

**(3)**

***Comment*:** Line 113-115: Only mention that the “Sites with diverse ground conditions were selected, including hard, medium and soft rocks/soils.”

***Section***: Materials and methods

***Response*:** The sentence was expressed in a better linguistic manner as recommended.

***Original manuscript:***

The evaluation target groups were sites that were constructed by applying the top-down construction method, and voluntarily agreed to the evaluation. The sites was selected so that sites with diverse types of ground, including soft rock, hard rock, and soil.

***Revised manuscript:***

Sites with diverse ground conditions were selected, including hard, medium and soft rocks/soils. ***(Manuscript Page 7, Line 112~113)***

**(4)**

***Comment*:** What do you mean by “S2, S3 and … Tables”?

***Section***: Materials and methods

***Response*:**

S2, S3 Tables are Supporting Information.

“S4 and S5 Tables” was changed by format citations

***Original manuscript:***

S 4 ~ 6 Tables

***Revised manuscript:***

S4 and S5 Tables ***(Manuscript Page 15, Line 221-222)***

**(5)**

***Comment*:** Line 454-456: Complete the sentence. It is linguistically incomplete with no punctuation at the end of the sentence. “Therefore, engineering and managerial improvement necessary to improve the working environment in underground excavation workshop”.

***Section***: Conclusions

***Response*:** The sentence was completed as recommended.

***Original manuscript:***

Therefore, engineering and managerial improvement necessary to improve the working environment in underground excavation workshop

***Revised manuscript:***

Therefore, engineering and managerial improvement is necessary to improve the working environment in underground excavation workshop ***(Manuscript Page 36, Line 444-446)***

**(6)**

***Comment*:** Line 461-464: Kindly change the sentence “Through this study, to improve the working environment and prevent occupational diseases in underground excavation workers, the results of the evaluation of the hazards were derived, and this data might be used for preparing engineering and managerial improvement measures. “ To

“This study was aimed to improve the working environment and prevent occupational diseases in underground excavation workers: The hazards are evaluated and the data might be useful for preparing safe engineering and managerial measures.”

***Section***: Conclusions

***Response*:** The sentence was expressed in a better linguistic manner as recommended.

***Original manuscript:***

Through this study, to improve the working environment and prevent occupational diseases in underground excavation workers, the results of the evaluation of the hazards were derived, and this data might be used for preparing engineering and managerial improvement measures

***Revised manuscript:***

This study was aimed to improve the working environment and prevent occupational diseases in underground excavation workers: The hazards are evaluated and the data might be useful for preparing safe engineering and managerial measures. ***(Manuscript Page 37, Line 454-456)***
